# A machine learning based two-stage clinical decision support system for predicting patients’ discontinuation from opioid use disorder treatment: retrospective observational study

**DOI:** 10.1186/s12911-021-01692-7

**Published:** 2021-11-26

**Authors:** Md Mahmudul Hasan, Gary J. Young, Jiesheng Shi, Prathamesh Mohite, Leonard D. Young, Scott G. Weiner, Md. Noor-E-Alam

**Affiliations:** 1 Department of Mechanical and Industrial Engineering, College of Engineering, Center for Health Policy and Healthcare Research, Northeastern University, 360 Huntington Avenue, Boston, MA 02135 USA; 2 D’Amore-McKim School of Business, Bouve College of Health Sciences, Center for Health Policy and Healthcare Research, Northeastern University, 360 Huntington Avenue, Boston, MA 02135 USA; 3 Prescription Monitoring Program, Massachusetts Department of Public Health, Boston, MA 02108 USA; 4 Department of Emergency Medicine, Division of Health Policy and Public Health, Brigham and Women’s Hospital, 75 Francis Street, NH-226, Boston, MA 02115 USA

## Abstract

**Background:**

Buprenorphine is a widely used treatment option for patients with opioid use disorder (OUD). Premature discontinuation from this treatment has many negative health and societal consequences.

**Objective:**

To develop and evaluate a machine learning based two-stage clinical decision-making framework for predicting which patients will discontinue OUD treatment within less than a year. The proposed framework performs such prediction in two stages: (i) at the time of initiating the treatment, and (ii) after two/three months following treatment initiation.

**Methods:**

For this retrospective observational analysis, we utilized Massachusetts All Payer Claims Data (MA APCD) from the year 2013 to 2015. Study sample included 5190 patients who were commercially insured, initiated buprenorphine treatment between January and December 2014, and did not have any buprenorphine prescription at least one year prior to the date of treatment initiation in 2014. Treatment discontinuation was defined as at least two consecutive months without a prescription for buprenorphine. Six machine learning models (i.e., logistic regression, decision tree, random forest, extreme-gradient boosting, support vector machine, and artificial neural network) were tested using a five-fold cross validation on the input data. The first-stage models used patients’ demographic information. The second-stage models included information on medication adherence during the early phase of treatment based on the proportion of days covered (PDC) measure.

**Results:**

A substantial percentage of patients (48.7%) who started on buprenorphine discontinued the treatment within one year. The area under receiving operating characteristic curve (C-statistic) for the first stage models varied within a range of 0.55 to 0.59. The inclusion of knowledge regarding patients’ adherence at the early treatment phase in terms of two-months and three-months PDC resulted in a statistically significant increase in the models’ discriminative power (p-value < 0.001) based on the C-statistic. We also constructed interpretable decision classification rules using the decision tree model.

**Conclusion:**

Machine learning models can predict which patients are most at-risk of premature treatment discontinuation with reasonable discriminative power. The proposed machine learning framework can be used as a tool to help inform a clinical decision support system following further validation. This can potentially help prescribers allocate limited healthcare resources optimally among different groups of patients based on their vulnerability to treatment discontinuation and design personalized support systems for improving patients’ long-term adherence to OUD treatment.

## Background

Opioid use disorder (OUD) is characterized as the problematic pattern of chronic opioid use including prescription opioid analgesics and illicit drugs (e.g., heroin or non-prescription fentanyl) [1]. An estimated 2.4 million Americans across all age groups and backgrounds are affected by OUD [2]. Furthermore, drug overdose deaths related to opioids have reached an epidemic level in the US, taking 136 lives every day [3]. In addition to the substantial death toll from this condition, OUD is also associated with various acute and chronic health conditions that results in emergency visits, hospitalizations, and impaired functional status [4–10]. Annually, the resulting estimated societal costs of OUD and opioid overdose exceeds $78 billion [6]. As such, reducing the prevalence of OUD and ensuring sustained treatment for this condition is a health policy issue of critical importance.

OUD can be treated with opioid agonist therapy that includes psychosocial therapy and medication. Buprenorphine/naloxone (hereinafter buprenorphine) is one of the three FDA approved evidence-based medication options for treating OUD. Individuals undergoing buprenorphine treatment typically progress through a series of phases: *(i) induction (up to one week), (ii) stabilization (up to 2 months), (iii) maintenance (*> *2 months), and (iv) possible discontinuation* [11]. Treatment often involves periods of relapse and remission*.* Patients with long periods of abstinence usually recover well from OUD; nevertheless, the risk of accidental overdose, suicide, trauma and infectious diseases never disappears [12]. As such, there is uncertainty regarding the suitable timing for discontinuing buprenorphine treatment and so optimal treatment duration remains controversial.

While the minimum duration for buprenorphine treatment is recommended to be a year or more, the American Society of Addiction Medicine recommends that lifetime use of buprenorphine should be the standard of care to avoid the risk of relapse to non-medical use of opioids [13]. Some evidence suggests that successful adherence to long-term buprenorphine treatment is associated with decreased non-medical use of opioids, reduced mortality, enhanced quality of life, and improved functional status [14–17].

However, retaining patients in treatment is a challenge as a large proportion of patients discontinue prematurely within the first few months [18–21]. Several studies have reported that at least 50% of patients enrolled in office-based buprenorphine treatment do not continue treatment for even 12 months [18, 20–25]. An individual’s adherence to buprenorphine treatment may vary due to various factors including socio-economic status, severity of addiction problem, and patients’ social support system [26, 27]. Determining the relative importance of these factors is a key consideration for improving patients’ adherence to buprenorphine treatment.

Studies focusing on predicting which patients are more likely to discontinue buprenorphine treatment prematurely are quite limited. Extant studies are based largely on regression-based techniques for investigating the influence of different patient- and provider-level factors on adherence and related outcomes of buprenorphine treatment [20, 25, 28–30]. However, previous studies largely focused on vulnerable populations such as Medicaid [30] and US Veterans [31], despite OUD being very prevalent throughout the US population. While a few studies [25, 28, 29] included commercially insured populations, they did not report the predictive accuracy of their models as the primary focus was to analyze associations of patient- and provider-level characteristics with treatment adherence. This potentially limits the applicability of these predictive models to different clinical settings.

As an alternative to regression-based techniques, other machine learning methods such as different variants of tree-based algorithms, support vector machine (SVM), and neural nets are commonly used for healthcare applications, primarily due to their ability to model complex interactions among predictors that are difficult, if not impossible, to model using regression. Although several studies [20, 25, 28–30] have used regression to analyze the association of different risk factors with treatment discontinuation, evidence on the application of other tree-based methods, SVM and neural nets for predicting premature discontinuation from OUD treatment is quite limited. Thus, the objective of this study was to develop and evaluate a machine learning based two-stage clinical decision-making framework for predicting which patients will discontinue treatment in less than a year of treatment initiation. Alongside regression, we also investigated the performance of other machine learning models for this decision-making framework. We primarily utilized patients’ demographic and prescription refill history from a large-scale healthcare administrative claims dataset for a statewide commercially insured population who was undergoing treatment for OUD with buprenorphine. The prediction occurred in two stages: (1) before initiating buprenorphine treatment, and (2) during the treatment phases of stabilization or early maintenance. The first stage model can potentially help prescribers leverage relatively little patient-level information that is available at the time of initiating treatment to judge whether a patient with OUD is a good candidate for buprenorphine treatment. The second stage model can enable prescribers to predict whether a patient is likely to be able to successfully complete the treatment once he/she has had some experience with this medication. We also constructed a set of decision rules for assessing whether a specific patient is likely to discontinue treatment. The proposed framework can be implemented in a clinical setting in the form of a *clinical decision support system* (CDSS) that can assist prescribers in designing targeted interventions for improving patient outcomes and allocating limited healthcare resources optimally among different groups of patients based on their vulnerability to treatment discontinuation.

## Methods

### Data sources

As a primary data source, we used administrative data from the Massachusetts All Payer Claims Database (MA APCD). The Center for Health Information and Analysis (CHIA) collects and maintains these de-identified data. The APCD includes completely adjudicated pharmacy, medical and dental claims for the majority of the Massachusetts’ populations enrolled in commercial insurance plans and Medicaid programs. This commercial database also includes claims for individuals covered by Medicare supplement policies and Medicare Advantage. However, claims for certain individuals covered by some health plans such as Federal Employees Health Benefit Plan, TRICARE, workers’ compensation, and Veterans Health Administration Plan are not included in APCD. Due to federal rules regarding patient privacy, in most cases state agencies assembling claims databases must remove codes related to substance use disorder including substance use disorder treatment [32]. We applied for and received from CHIA a waiver to this policy subject to the removal of certain patient-level information regarding residence that potentially could be used to identify specific patients.

For this retrospective and longitudinal study, we primarily used APCD pharmacy claims and member eligibility data for commercially insured individuals between years 2013 and 2015. The pharmacy claims dataset records the patients' history of filling prescription medications that are reimbursed by commercial insurers. Each claim includes information about a patient's age, gender, name of the pertinent drugs and associated national drug codes (NDC), quantity and days for which the drugs were prescribed, and prescriber (including name, national provider identifier, county) who issued the prescription. From the pharmacy claims dataset, we used member age reported at the time of focal buprenorphine prescription, member gender, NDC codes associated with buprenorphine medication, date the prescription was filled, and days supply of the medication. Patients’ clinical information was obtained from the principal diagnoses recorded in the medical claims datasets. These diagnoses were coded using internal classification of diseases ninth revision (ICD-9) codes. For information about the prescriber who issued the buprenorphine prescription, we used the national provider identification (NPI) number and the county where the prescriber’s practice was located. To obtain information on the prescriber specialty and additional training relevant to addiction treatment, we first interlinked APCD pharmacy claims data with National Plan and Provider Enumeration Systems (NPPES) dataset using provider NPI number. Using the extracted taxonomy codes from the NPPES dataset, we identified prescriber specialty from the healthcare provider taxonomy code sets provided by National Uniform Claim Committee.

### Study design and sample

We included patients who met the following three inclusion criteria: (1) started treatment for OUD with buprenorphine between January and December 2014, and did not appear to have any other buprenorphine prescription one year prior to the first prescription that was recorded in 2014, (2) aged 18 years or older at the time of that first buprenorphine prescription, and (3) Massachusetts resident and continuously insured by any commercial health plan from January 2013 to December 2015. The third criterion was adopted to ensure that discontinuation was not the result of individuals losing their health insurance or leaving Massachusetts as the state of residence. The NDCs were used to search for the claims related to buprenorphine prescriptions. For the second criterion, we followed previous research [20, 30, 33] by identifying individuals for whom a prescription claim was filed for buprenorphine at any time from January to December 2014, but did not have any records of previous claims for this medication for at least 12 months. We defined the date at which the first buprenorphine claim was recorded within the relevant time period as an index date for the treatment.

### The two-stage machine learning framework for the prediction of treatment discontinuation

As noted, we developed and tested a unique two-stage machine learning framework to predict which patients are at a substantially higher risk of premature discontinuation from OUD treatment. The first stage is defined as the point in time at which a prescriber and a patient consider whether buprenorphine is a good treatment option for the patient and what challenges the patient may face in achieving good medication adherence. This is also when prescribers will typically inform patients of the potential risks, benefits, and alternative treatment options for OUD. In considering this decision, the prescriber will typically know something about a patient’s demographic background and clinical history, including any previous substance use disorder, information that is potentially helpful for predicting a patient’s medication adherence.

Because patients’ poor treatment adherence may lead to adverse outcomes, it is important for the prescriber to assess and reassess periodically a patient's response to and motivation for treatment during the early treatment phase [27]. Thus, the second stage decision, which occurs between two or three months from the time of treatment initiation, provides an opportunity for the prescriber and patient to assess the patient’s treatment progress. A critical piece of additional information at this point is a patient’s prescription refill history that can be used as a proxy measure for actual medication adherence to date [25, 28, 29]. While in the past prescribers would often lack such information, advances in the integration of clinical and pharmacy data and access to prescription drug monitoring data are making such information readily available to prescribers. With this additional information, the prescriber is in a better position to predict a patient’s likelihood of discontinuing treatment. More importantly, if a patient appears to be at a high risk of treatment discontinuation, the prescriber has an opportunity to help put in place support systems the patient may need to remain in treatment and achieve a positive outcome.

**Fig. 1:**
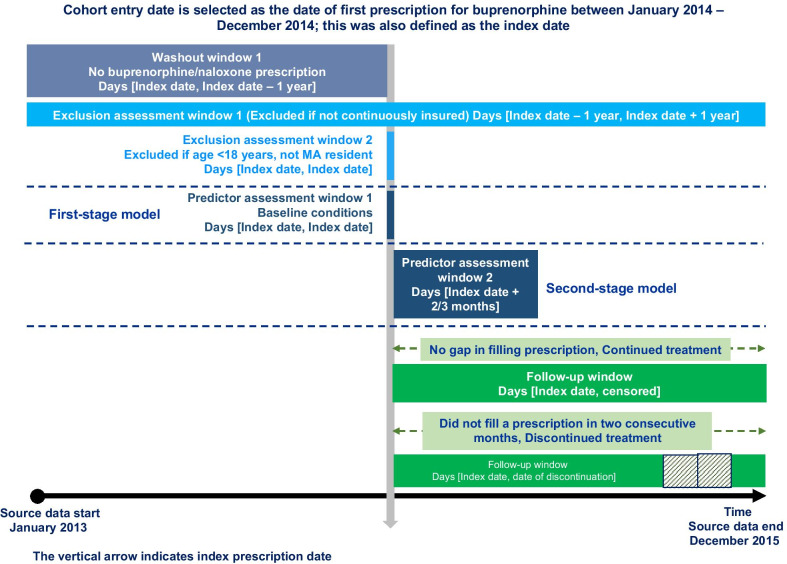
Conceptual framework for the study design

**Fig. 2:**
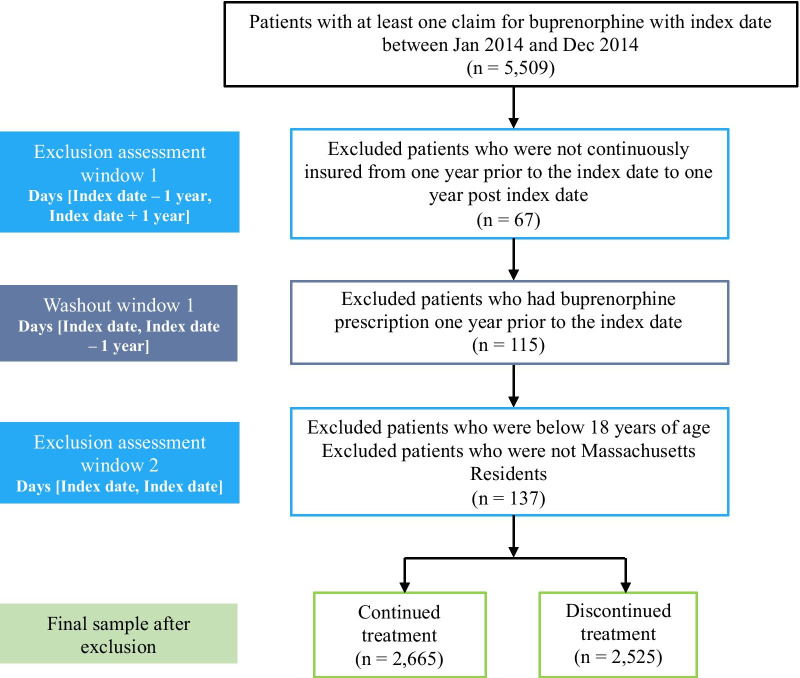
Sample selection flow chart

We framed this decision as a binary classification problem. We considered discontinuation from buprenorphine treatment within one year of treatment initiation as the outcome for the machine learning models. For each eligible patient, we investigated his/her longitudinal prescription claims history for up to one year from the index date to determine if, and when, discontinuation occurred for buprenorphine treatment. Consistent with prior studies [20, 25, 30, 34–36], we defined treatment discontinuation as an apparent gap in patients’ prescription refill patterns for buprenorphine. Specifically, a patient was considered as discontinuing treatment if there existed a gap of 60 consecutive days within which he/she did not appear to fill a prescription for buprenorphine. Patients without such a gap of 60 consecutive days within the pre-defined one year follow up period were considered as continuing the treatment (as known as right censored). Drawing from previous research [37], we present this conceptual framework for designing the study in Fig. 1. Based on the three above-mentioned inclusion/exclusion criteria, a total of 5190 patients were retained in the final study sample. In Fig. 2, we present the number of patients who were excluded in each of these steps and the final study sample with patients’ treatment continuation/discontinuation status.

For the proposed two-stage machine learning framework, we considered several patient-level characteristics that can be used as features to predict treatment discontinuation. The first-stage prediction accounted for patient-level demographic characteristics that would be available to the prescribers at this stage. Specifically, for the patient-level characteristics, we included patients’ age, gender, types of commercial health insurance, and a proxy for socio-economic background. We classified patients into three groups based on the type of commercial insurance coverage: Health Maintenance Organization (HMO), Preferred Provider Organization (PPO) and other (which primarily comprises patients with indemnity coverage). Because our dataset did not include information pertaining to patient residence, we used the county in which a patient’s primary prescriber was located as the patient’s residing county. We selected this as a proxy after conducting an analysis on a similar claims dataset that did not include substance use disorder codes but did include information on patient residence. We found that the great majority of patients were under the care of a prescriber located in the same county where the patient resided. We identified the patient’s primary prescriber as the prescriber from whom a patient received the majority of the buprenorphine prescriptions during the relevant study time frame.

We determined the urbanicity of a patient’s assigned county (i.e., whether a county is urban or rural) and its median household income. To determine urbanicity of the counties in Massachusetts, we followed the guidelines of the Office of Rural Health Policy (ORHP) [38]. According to these guidelines, any counties that are not designated as parts of Metropolitan Areas by the Office of Management and Budget (OMB) are considered rural. The ORHP designates Dukes, Franklin, and Nantucket as rural counties in Massachusetts. However, despite being designated as a metropolitan county (i.e., urban county), some counties may include areas that are largely rural. To rectify this, the ORHP developed rural urban commuting area codes (RUCAs) to designate rural areas within a designated metropolitan county for the purpose of awarding rural health grants [38]. In compliance with these guidelines, we found that Barnstable, Berkshire and Worcester counties in Massachusetts have some areas that are designated as rural [38, 39]. Given that the proportion of rural areas in Barnstable and Worcester counties are low compared to the urban areas [39], we considered Barnstable and Worcester as urban counties. However, because a significant proportion of Berkshire county is designated as rural, we considered Berkshire as a rural county in our study.

We approached the potential predictive value of patients’ medical history with the following two considerations. First, we did not have complete information regarding the medical history for all patients in the sample due to missing patient-level identifiers within medical claims file. We lacked this information for approximately 40% of the sample. Second, we had concerns about the reliability of the most pertinent clinical information regarding history of substance use disorder as some clinicians are reluctant to document such information in the medical claims of patients [40, 41]. Accordingly, we initially developed and tested our two-stage model without patients’ baseline clinical information and then subsequently conducted a sensitivity analysis for including clinical information.

For this two-stage model, we also included several characteristics of the patient’s primary prescriber pertinent to specialty and training. Specifically, we used three categories for physician specialty: (i) family or internal medicine, (ii) psychiatry, and (iii) other specialty. We further included two binary predictors for whether or not the primary prescriber (i) had formal training in addiction treatment, and (ii) was a pain specialist.

For the second stage, we included patients’ early sign of treatment adherence (stabilization and early maintenance phase) alongside the other patient-level characteristics. To determine patients’ treatment adherence during the early treatment phase, we computed proportion of days covered (PDC). The PDC is defined as the proportion of days a patient had buprenorphine medication on hand within a given time frame. In particular, we measured the PDC within the first two months and the three months following treatment initiation. We included PDC with their actual values and separately evaluated our proposed machine learning framework using two months and three months PDC. As a sensitivity analyses, in line with existing studies [28, 29], we also dichotomized PDC based on a threshold of 80% or higher to determine patients’ adherence (i.e., low or high) at early treatment phase.

### Machine learning methods and prediction performance evaluation

Our primary goal was to predict buprenorphine treatment discontinuation in two stages of treatment, and a secondary goal was to construct a set of interpretable and actionable decision classification rules to help clinical decision making. We used five-fold cross validation to train and test the models. For both first and second stage prediction, we used six well-known machine learning algorithms: logistic regression, decision tree, random forest, extreme gradient boosting, support vector machine, and artificial neural network. All these algorithms were evaluated in the scikit-learn machine learning library in python 3.0 software. We performed an exhaustive search over a grid of pre-selected hyper-parameter values for each model. These grids of hyper-parameters were optimized by a five-fold cross validated grid search method with recall set as a scoring method. This set of hyper-parameters alongside the parameters that were selected for the final model implementation are listed in Table 3 in Appendix. We evaluated the performance of these algorithms using the area under the receiving operating characteristic curve (ROC). Alongside ROC curve, we also reported area under precision-recall curve. In addition, we computed other performance metrices such as precision, recall, and F1-score for each machine learning model. For each performance metric, we reported the mean and standard deviation of the five values that were obtained in each iteration of the five-fold cross validation.

## Results

The study sample comprised 5190 patients. We present the baseline patient-level demographic and prescriber-level characteristics for the entire sample in Table 1. The average age of the patients was 36.4 ± 11.3 years. The mean PDC were 88% and 84.3% within the first two and three months following treatment initiation, respectively. After dichotomizing the PDC measure, we observed that the percentage of patients with high values of PDC within the first two months and three months was 81.1 and 75.6, respectively.

**Table 1:** Characteristics of Massachusetts commercially insured patients with buprenorphine prescriptions and by treatment discontinuation^a^ status

	Total	Continued	Discontinued	RR (95% CI)	*P *value
Number of patients	5190	2665	2525		
Patient characteristics					
Patient age	36.4 ± 11.3	37.3 ± 10.9	35.6 ± 11.5	N/A	< 0.001
Patient income	72,109 ± 13,087	71,581 ± 12,961	72,667 ± 13,199	N/A	0.003
Patient gender					
Male	3165 (61)	1596 (59.9)	1569 (62.1)	0.95(0.89, 1.01)	0.09
Female	2025 (39)	1069 (40.1)	956 (37.9)	Reference	Reference
Patient location					
Urban	3582 (69)	1793 (67.3)	1789 (70.9)	Reference	Reference
Rural	1608 (31)	872 (32.7)	736 (29.1)	0.92 (0.86, 0.97)	0.005
Insurance type					
HMO	1212 (23.4)	521 (19.5)	691 (27.4)	1.27 (1.19, 1.35)	< 0.001
PPO	427 (8.2)	187 (7)	240 (9.5)	1.25 (1.14, 1.37)	< 0.001
Others	3551 (68.4)	1957 (73.5)	1594 (63.1)	Reference	Reference
Provider specialty					
Primary care	2951 (56.9)	1537 (57.7)	1414 (56)	0.93 (0.87, 0.99)	0.06
Psychiatry	1055 (20.3)	553 (20.8)	502 (19.9)	0.92 (0.85, 1.01)	0.04
Others	1184 (22.8)	575 (21.5)	609 (24.1)	Reference	Reference
Addiction treatment specialist					
No	4831 (84.4)	2481 (93.1)	2350 (93.1)	Reference	Reference
Yes	359 (15.6)	184 (6.9)	175 (6.9)	1.00 (0.90, 1.12)	0.97
Pain specialist					
No	5114 (98.5)	2633 (98.8)	2481 (98.3)	Reference	Reference
Yes	76 (1.5)	32 (1.2)	44 (1.7)	1.19 (0.98, 1.44)	0.11
Patients’ early treatment adherence					
Patients’ PDC within first two months (continuous measure)	88.0 ± 25.6	95.3 ± 16.7	80.3 ± 30.6	N/A	< 0.001
Patients’ PDC within first three months (continuous measure)	84.3 ± 28.9	94.2 ± 19.0	73.9 ± 33.6	N/A	< 0.001
PDC within first two months					
High ($\ge$ 80%)	4209 (81.1)	2459 (92.3)	1750 (69.3)	Reference	Reference
Low ($<$ 80%)	981 (18.9)	206 (7.7)	775 (30.7)	1.90 (1.81, 1.99)	< 0.001
PDC within first three months					
High ($\ge$ 80%)	3924 (75.6)	2424 (91)	1500 (59.4)	Reference	Reference
Low ($<$ 80%)	1266 (24.4)	241 (9)	1025 (40.6)	2.11 (2.01, 2.22)	< 0.001

^a^Treatment discontinuation was defined as a gap of 60 consecutive days without a buprenorphine prescription

Differences were calculated for the patient-level characteristics with continuous measures using p-values computed from a t-test

Table 1 also presents descriptive statistics for patients stratified by treatment continuation/discontinuation status. A total of 2525 patients (48.7%) discontinued treatment based on a gap of 60 consecutive days without a buprenorphine prescription during the one-year treatment period. Thus, a significant proportion of patients discontinued the treatment well before sustained remission. The average age and PDC during the first two and three months of treatment initiation for the patients who discontinued treatment were less than those who continued (*p *value < 0.05). Table 1 also shows the risk ratios (RR) and associated p-values for the risk of treatment discontinuation that we computed for different patient- and prescriber-level characteristics. Among those with high PDC (i.e., 80% or higher) during the first two and three months following treatment initiation, 41.5% and 38.3% (not directly shown in table) discontinued treatment, respectively. By contrast, among patients with low PDC at the first two and three months following treatment initiation, 79% and 80.9% discontinued treatment within the first year of treatment initiation, respectively. Patients with low PDC had 1.9 (95% CI = 1.81–1.99, *p *value < 0.001) and 2.11 (95% CI = 2.01—2.22, *p *value < 0.001) times the risk of treatment discontinuation compared to those who had high PDC during the first two and three months following treatment initiation, respectively, indicating that patients’ low adherence in the early treatment phase is highly associated with a poor treatment outcome. Patients whose insurance plan type was HMO (RR = 1.27, 95% CI = 1.19—1.35, *p *value < 0.001) or PPO (RR = 1.25, 95% CI = 1.14—1.37, *p *value < 0.001) had increased risk for treatment discontinuation compared to those who had indemnity insurance.

**Fig. 3:**
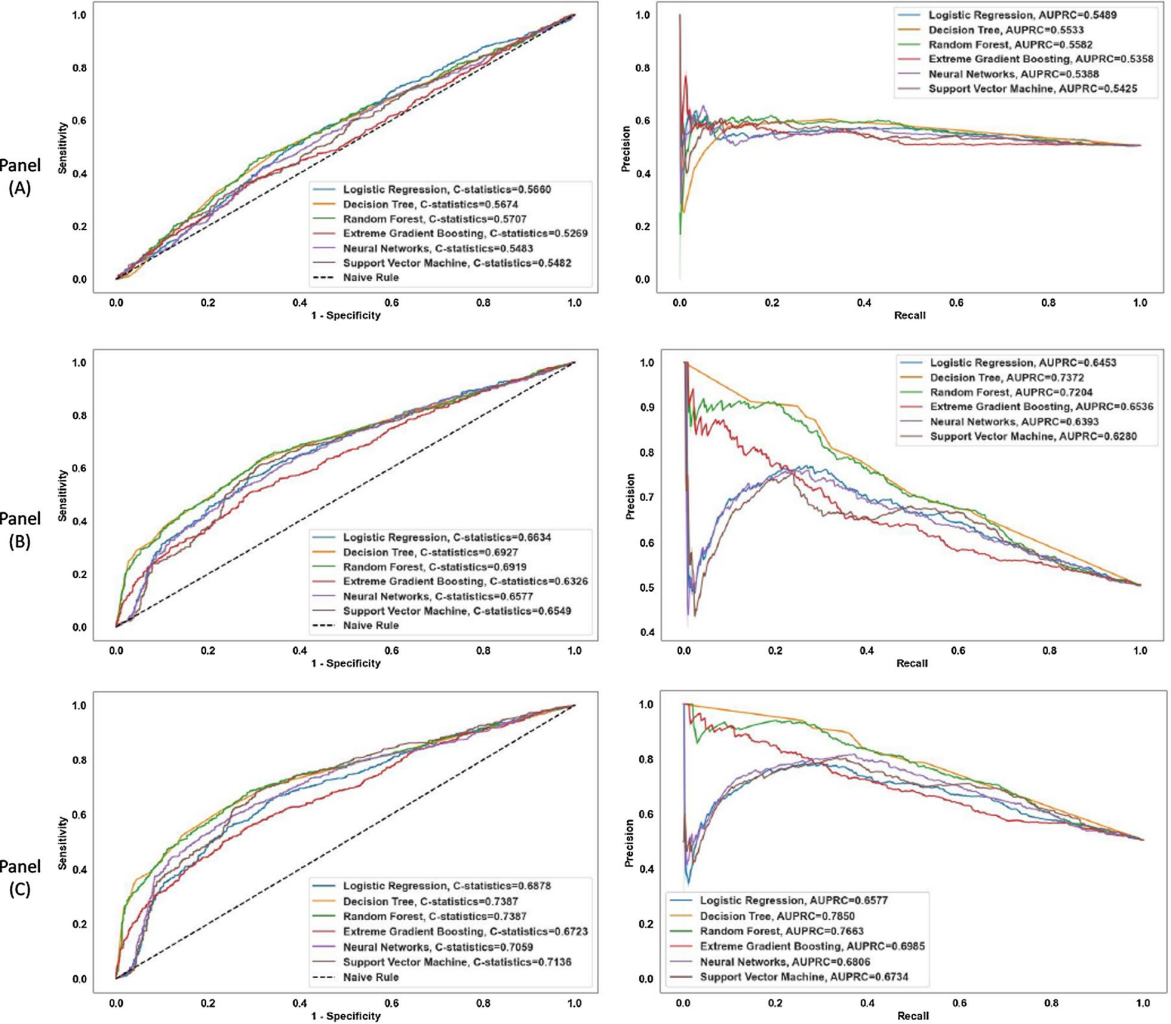
ROC curve (left) and precision-recall curve (right) for the 1st-stage model including baseline predictors (Panel **A**); ROC curve (left) and precision-recall curve (right) for the 2nd-stage model including baseline predictors and two-months PDC as a continuous variable (Panel **B**); ROC curve (left) and precision-recall curve (right) for the 2nd-stage model including baseline predictors and three-months PDC as a continuous variable (Panel** C**)

Table 2 presents the predictive performance (i.e., precision, recall, F1-score, C-statistic) of selected machine learning models in two stages that included PDC as a continuous measure for the second-stage models. The ROC and precision-recall curves of all the models used in two stages are presented in Fig. 3. For PDC as a dichotomous variable, the models' performance metrices as well as ROC and precision-recall curves from sensitivity analyses are presented in Table 4 and Fig. 6 in the Appendix, respectively. Alongside recall, we used the area under the ROC curve, alternatively known as the C-statistic, to evaluate the performance of these models.

**Table 2:** Buprenorphine treatment discontinuation prediction performance of machine learning models at first and second stage of treatment

Treatment stage for making prediction	Performance metrices	Decision tree	Random forest	Extreme gradient boosting	Logistic regression	Neural network	Support vector machine
First stage models with baseline predictors	Precision	0.57 ± 0.01	0.56 ± 0.02	0.53 ± 0.02	0.56 ± 0.02	0.56 ± 0.04	0.57 ± 0.03
	Recall	0.44 ± 0.02	0.49 ± 0.02	0.52 ± 0.03	0.38 ± 0.02	0.42 ± 0.05	0.38 ± 0.03
	F1 score	0.49 ± 0.02	0.53 ± 0.02	0.52 ± 0.03	0.45 ± 0.01	0.47 ± 0.03	0.38 ± 0.02
	C-statistics	0.58 ± 0.01	0.59 ± 0.02	0.55 ± 0.02	0.57 ± 0.02	0.57 ± 0.02	0.56 ± 0.02
Second stage models with 2 months PDC* and baseline predictors	Precision	0.67 ± 0.02	0.68 ± 0.01	0.59 ± 0.02	0.69 ± 0.03	0.67 ± 0.06	0.66 ± 0.03
	Recall	0.54 ± 0.04	0.55 ± 0.03	0.59 ± 0.03	0.46 ± 0.02	0.46 ± 0.05	0.51 ± 0.01
	F1 score	0.60 ± 0.03	0.61 ± 0.02	0.59 ± 0.02	0.55 ± 0.02	0.58 ± 0.01	0.58 ± 0.01
	C-statistics	0.69 ± 0.01	0.71 ± 0.01	0.65 ± 0.02	0.68 ± 0.02	0.67 ± 0.02	0.68 ± 0.02
Second stage models with 3 months PDC* and baseline predictors	Precision	0.69 ± 0.02	0.71 ± 0.02	0.62 ± 0.02	0.74 ± 0.03	0.73 ± 0.05	0.72 ± 0.07
	Recall	0.62 ± 0.04	0.60 ± 0.03	0.63 ± 0.04	0.40 ± 0.01	0.50 ± 0.04	0.49 ± 0.07
	F1 score	0.65 ± 0.03	0.65 ± 0.03	0.62 ± 0.03	0.40 ± 0.01	0.58 ± 0.02	0.58 ± 0.04
	C-statistics	0.74 ± 0.02	0.75 ± 0.03	0.69 ± 0.03	0.70 ± 0.02	0.71 ± 0.02	0.72 ± 0.03

*PDC is included in the model as a continuous variable

Each performance metric is reported as mean and standard deviation of the five values obtained from each of the five folds of cross validation

The C-statistic of the first-stage models with only baseline patient-level predictors varies within a range of 0.55 to 0.59. While these values are not indicative of high predictive power, inclusion of information about medication adherence in the form of PDC resulted in a statistically significant increase in the models’ ability to predict treatment discontinuation (p-value < 0.001). Specifically, we found that the models’ C-statistic lies within a range of 0.65 to 0.71 when two-months PDC were included alongside other baseline patient-level characteristics. Similarly, inclusion of three-months PDC, in addition to other patient-level features, increased the models’ C-statistic further, between 0.69 and 0.74. The highest increase in C-statistic was observed for the decision tree model. Specifically, the C-statistic for this model increased from 0.58 to 0.69 and to 0.74 (i.e., increased 19% and 27.5%) for the models including two-months and three-months PDC, respectively. Such increases in the recall value of the decision tree model was higher than the increase observed for the C-statistic. An increase in the recall value from 0.44 to 0.54 and 0.62 (i.e., increased roughly 23% and 41%) was observed for this model after including two-months and three-months PDC, respectively. Considering both recall and C-statistic, we observed comparable performance for the decision tree, random forest, and gradient boosting models. The predictive performance of the selected models that included PDC as a dichotomous variable was also comparable (Table 4 in Appendix). In addition, the C-statistic and recall value of the decision tree model also increased in a similar way after including two-months and three-months PDC as dichotomous variables.

**Fig. 4:**
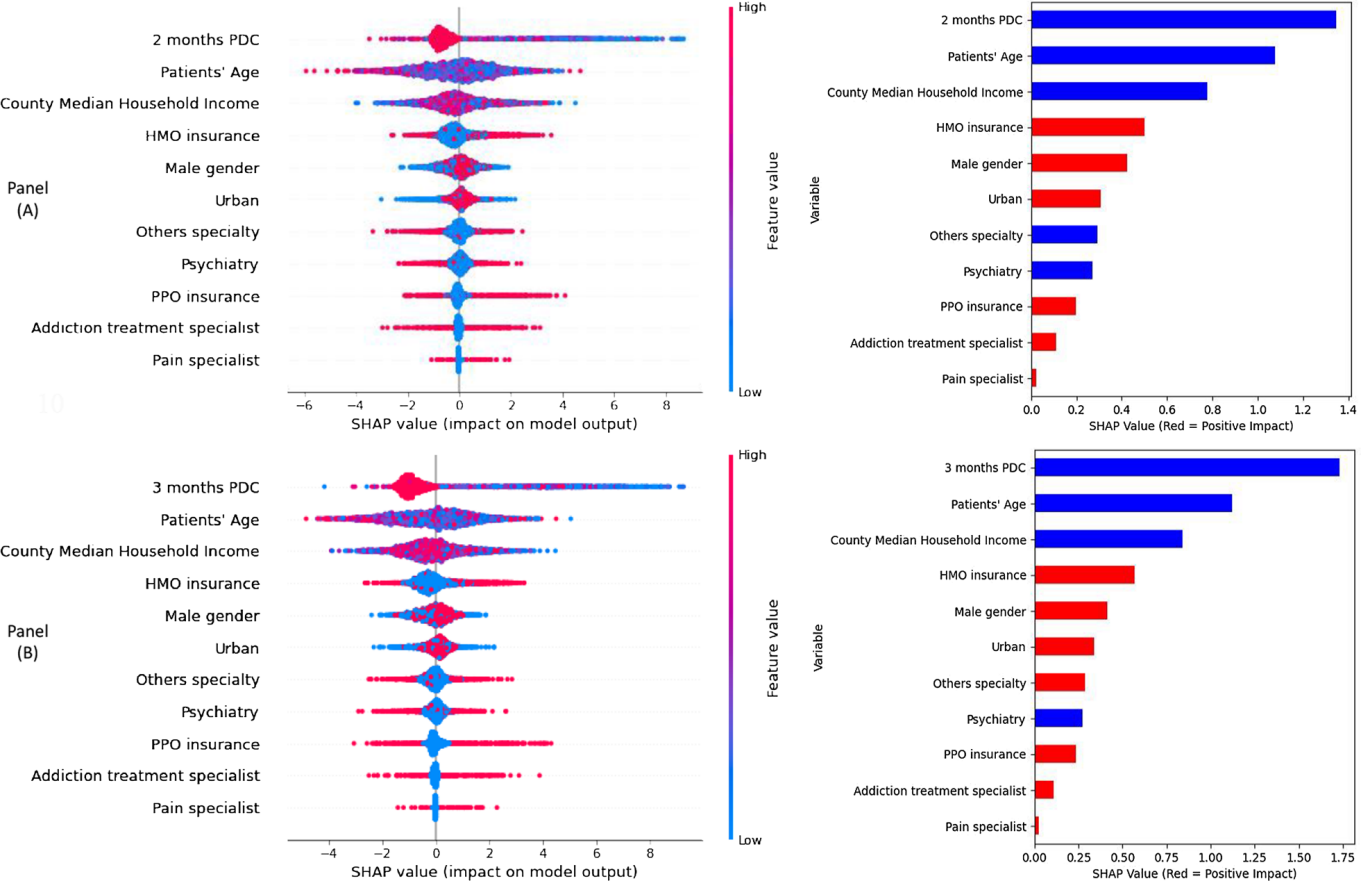
Variable importance plots using SHAP values from extreme gradient boosting model including two-months PDC (Panel **A**) and three-months PDC (Panel **B**) as a continuous measure; SHAP value computed from individual feature’s values and their impact (both positive and negative) on treatment discontinuation (left in Panel **A** and Panel **B**); Average SHAP values of features showing average impact on and correlation with treatment discontinuation (right in Panel **A** and Panel **B**)

Figure 4 shows two types of variable importance plots using SHAP values obtained from the gradient boosting models that included two-months (Panel A in Fig. 4) and three-months (Panel B in Fig. 4) PDC as continuous measures. Similar SHAP value plots for gradient boosting models including PDC as dichotomous variables are presented in Fig. 7 in Appendix. We observed similar patterns in variable importance (both in the order of features and their impact) for predicting treatment discontinuation including two-months and three-months PDC. The left plots in both Panel A and Panel B in Fig. 4 show the features in descending order of their importance, positive and negative impact of the individual values of different features with treatment discontinuation, the original value of a certain feature (indicated by red and blue color), and the impact of a certain feature’s value (i.e., whether the value is associated with higher or lower prediction) on treatment discontinuation. For example, a high value (indicated by red color) of the patients’ PDC during the first two-months and three-months following treatment initiation has a negative impact on the treatment discontinuation. The right plots in both Panel A and Panel B in Fig. 4 show the average impact of different features listed in descending order of their importance on treatment discontinuation. In addition, these plots also show for these features whether they were correlated positively (indicated by red color) or negatively (indicated by blue color) with treatment discontinuation. For example, we observed that PDC during the early treatment phase (i.e., during first two months and three months following treatment initiation) was negatively correlated with treatment discontinuation (i.e., the higher the PDC value is, the less likely it is for the patients to discontinue treatment). Although similar negative correlations were observed for patients’ age and median household income (based on their assigned county) with respect to treatment discontinuation, we observed that if a patient was male then he/she was more likely to discontinue treatment (i.e., a positive correlation). Accordingly, while PDC in early treatment phase remains as the most important feature for predicting treatment discontinuation, we also observed some degree of importance for patients’ age, gender, and median household income in their assigned county, and their insurance status.

As noted, we performed a sensitivity analysis for our two-stage model that included clinical information from patients’ medical records. We included in this analysis any codes pertaining to substance use disorder. We also assessed the predictive value from adding other types of clinical information to the models including selected mental health conditions (e.g., bipolar disorder, depression, and general anxiety) and overall disease burden. For overall disease burden, we used the Charlson comorbidity index that was computed based on information one year prior to the index date. This index is based on a list of nineteen conditions, which are identified using International Classification of Diseases (ICD) diagnoses codes from hospital abstract or physician billing claims data [42, 43]. The final index score is the weighted sum derived for all identified conditions, each of which has an assigned weight from one to six. A higher score indicates a greater level of overall disease burden. However, the inclusion of this information actually resulted in somewhat lower C-statistics for the models than those for the models based on the full sample, a result that most likely can be attributed to the smaller sample size. The key predictor of treatment discontinuation continued to be early phase medication adherence as measured by PDC. Accordingly, for the second goal of our investigation we relied on the results from the models that were informed with patients’ demographic and prescriber-level characteristics, and PDC.

The secondary goal of this study was to combine patient-level information from the first and second stage to formulate decision classification rules for predicting treatment discontinuation. Given that the C-statistic and recall of all the decision tree and random forest models were comparable, we chose the decision tree model due to its ease of interpreting the interactions among predictor candidates with respect to outcome. The decision tree utilized recursive partitioning to identify patients’ subgroups that are as homogeneous as possible with respect to the treatment discontinuation. Tracing the tree from the top feature in the first partition to the terminal feature (node) can uncover the interactions among independent features within the study cohort. Revealing these interactions can potentially help prescribers identify patients who may require additional support while they are undergoing treatment with buprenorphine to reduce the risk of premature discontinuation.

**Fig. 5:**
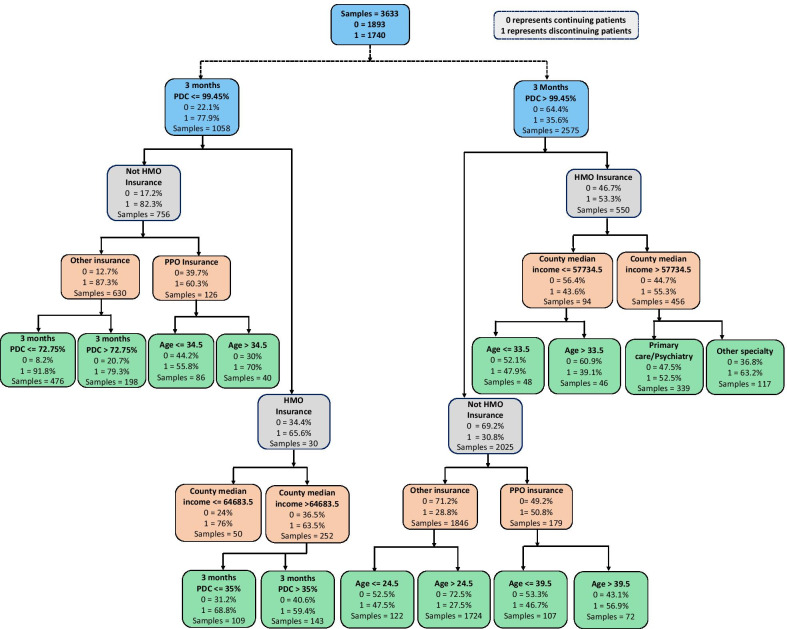
Decision classification rules for the prediction of treatment discontinuation using decision tree model (PDC considered as a continuous variable)

**Fig. 6:**
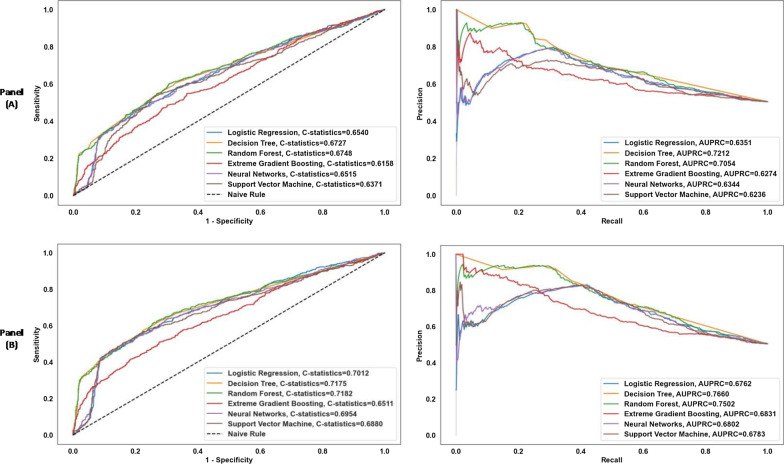
ROC curve (left) and precision-recall curve (right) for the 2nd-stage model including baseline predictors and two-months PDC as dichotomous variable (Panel A); ROC curve (left) and precision-recall curve (right) for the 2nd-stage model including baseline predictors and three-months PDC as a dichotomous variable (Panel B)

**Fig. 7:**
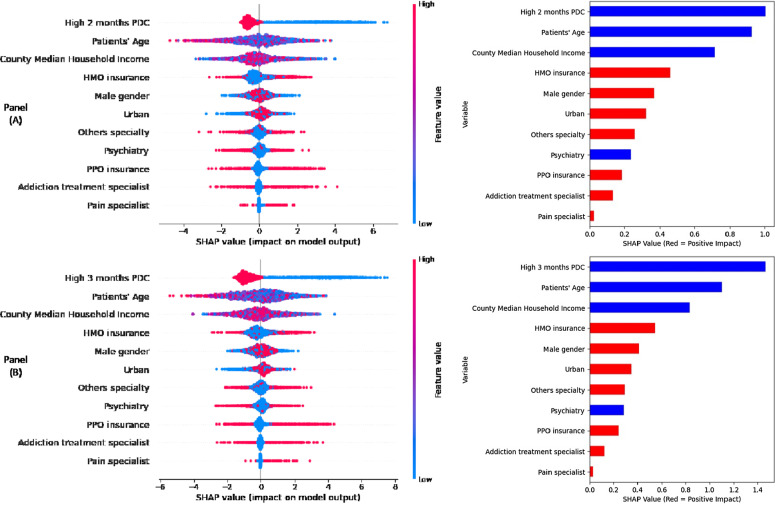
Variable importance plots using SHAP values from extreme gradient boosting model including two-months PDC (Panel **A**) and three-months PDC (Panel **B**) as a dichotomous variable; SHAP value computed from individual feature’s values and their impact (both positive and negative) on treatment discontinuation (left in Panel **A** and Panel **B**); Average SHAP values of features showing average impact on and correlation with treatment discontinuation (right in Panel **A** and Panel **B**

**Fig. 8:**
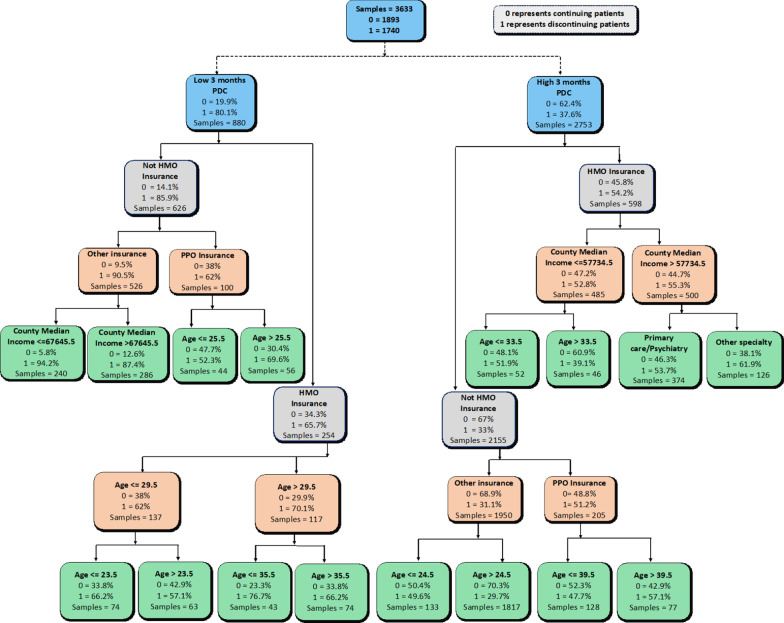
Decision classification rules for prediction of treatment discontinuation using decision tree model (PDC considered as a dichotomous variable)

We present the decision trees for the second-stage models that included three-months PDC in Figs. 5 and 8 (Fig. 8 presented in Appendix). Figure 5 shows the recursive partitioning created by the decision tree model that included three-months PDC as a continuous variable, and Fig. 8 (presented in Appendix) shows the recursive partitioning from the sensitivity analyses that included three-months PDC as a categorical variable (i.e., low and high three-months PDC). Similar trees can be constructed for the second stage models that included two-months PDC as a continuous as well as a dichotomous variable.

The decision trees started partitioning the data using two-months and three-months PDC to identify significant subgroups. Figures 5 and 8 show the data partitions beginning with three-months PDC. Each node shows the individual feature considered for the partition, the percentages of patients who continued and discontinued the treatment, and the total number of patients. The best classification identified by recursive partitions is shown in the terminal nodes. Nodes with significantly higher proportions of either continued or discontinued patients demonstrate the model’s higher confidence in achieving better predictive performance. For example, the decision tree model was able to identify one of the terminal nodes in Fig. 5 with 91.8% patients who discontinued the treatment, indicating a significant improvement in the model’s prediction performance over 77.9% identified in the first partition based on three-months PDC.

Figure 5 also shows that 77.9% patients with PDC less or equal to 99.45% in first three months of treatment initiation discontinued treatment within the first year. Patients in this group were first partitioned into subgroups based on their insurance status. Approximately 66% of patients with HMO insurance discontinued treatment. We observed that socio-economic status (median household income based on assigned county) alongside PDC and insurance status were significant predictors of treatment discontinuation. For the group of patients residing in areas with income approximately less or equal to $64,600, 76% discontinued treatment while 63.5% of the patients residing in areas with income higher than $64,600 discontinued treatment. Patients residing in the areas with income higher than approximately $64,600 were further partitioned based on their PDC in first three-months following treatment initiation. We observed that treatment discontinuation appeared to be higher for patients with PDC less than 35% than those whose PDC lies in between 35% and 99.45%. For the patients who did not have HMO insurance and had three-months PDC less or equal to 99.45% recursive partitioning revealed interactions among patients’ age, PDC, and insurance type. The majority of patients in this subgroup had indemnity insurance. After accounting for insurance status, patients with PDC less or equal to 72.75% were more likely to discontinue treatment than those with PDC higher than 72.75%. Similarly, variation in treatment discontinuation for subgroups created by recursive partitioning from the decision tree model including three-months PDC as a dichotomous variable (see Fig. 8 in Appendix), and two-months PDC as a continuous and  a dichotomous variable can be explained.

Using the recursive partitioning created by the second-stage models it is possible to formulate a set of decision classification rules that can show the probability of treatment discontinuation for patients with a certain profile. We provided the following classification rules as examples that are constructed from the decision tree model including first three-months PDC as a continuous variable:

- Classification Rule 1: If a patient has a PDC less than or equal to 35% at three months following treatment initiation, a PPO plan, and resides in areas where median household income is greater than $64,683, then the probability of discontinuing treatment is approximately 69%.
- Classification Rule 2: If a patient has a PDC less than or equal to approximately 72% at three months following treatment initiation, and an  indemnity insurance, then the probability of discontinuing treatment is over 91%.
- Classification Rule 3: If a patient has a high PDC (i.e., higher than 99%) at three months following treatment initiation, indemnity insurance, and is older than 24 years, then the probability of discontinuing the treatment is approximately 27%.
- Classification Rule 4: If a patient has a high PDC (i.e., higher than 99%) at initial three months following treatment initiation, HMO plan, resides in areas with income higher than $57,734, and received treatment from a provider who are not a primary care and psychiatrist, then the probability of discontinuing the treatment is approximately 63%.

From the sensitivity analyses that included PDC as a dichotomous variable, we observed largely similar patterns of treatment discontinuation for different subgroups identified by recursive partitioning. Although including PDC information as a continuous measure provides for specific thresholds, we observed that dichotomizing this PDC information using a threshold of 80% produced sub-classifications that are more interpretable from the clinical perspective without much of a loss in model predictive and discriminative power. We also observed that while PDC during the early treatment phase is a key predictor for treatment discontinuation, the decision tree model revealed potentially important interactions among other patient-level demographic and prescriber-level factors. While the probability of discontinuation is high generally for patients with lower PDC (i.e., PDC less than 80%) value during the early phase of treatment, we still see that when this information is combined with other patient-level characteristics, the probability of treatment discontinuation can vary substantially with a range of more than 42 percentage points. By contrast, a higher value of medication adherence during the first two and three months of treatment initiation is generally indicative of a much lower likelihood of treatment discontinuation. However, despite having a high PDC during the early treatment phase, the probability of treatment discontinuation can be as high as 62%. This pattern largely remained similar for the recursive partitioning that was created from the decision tree model including PDC as continuous measures.

## Discussion and conclusion

Our study proposed a two-stage machine learning framework to help prescribers predict patients’ premature discontinuation from OUD treatment and thus potentially help optimize the intended outcomes of buprenorphine treatment for patients with OUD. We successfully applied several machine learning models in two stages of patients’ buprenorphine treatment. Alongside logistic regression, we evaluated other machine learning methods and compared their predictive accuracy using data for a statewide commercially insured population. These models accounted for patient- and prescriber-level information that are known a priori to initiating treatment (i.e., first stage) and also patient-level information that can be acquired during the early phase of treatment (i.e., second stage) with buprenorphine.

For this particular study cohort, the C-statistic for the selected predictive models were comparable. This finding suggests the following: (1) any one of these predictive models could be selected to distinguish patients based on their treatment discontinuation status for this particular data and features set, and (2) the specific assumptions for different models used in this study did not have a quantifiable impact on the models’ ability to discriminate between patients based on whether or not they discontinue treatment. However, when the models’ recall values are considered in combination with the C-statistic, we observed that tree-based models outperform other models.

The C-statistic for the first-stage models did not achieve a very high value. However, this limitation of the machine learning models may well be explained from the standpoint of the models’ applicability in a clinical setting. Since our goal was to utilize the patient-level information that is readily available from a simple patient interview before starting treatment, models’ inputs were limited to a very few features entailing only patients’ demographic characteristics in the first-stage model.

While the first-stage models did not achieve high C-statistic, inclusion of information about the patients’ medication adherence in the early treatment phase improved the model’s predictive performance. This improvement in predictive performance was evident for all the machine learning models used in this study. This indicates that combining information about patients’ adherence at an early treatment stage with baseline patient- and prescriber-level information can provide valuable insights for designing targeted interventions to improve patients’ long-term adherence to OUD treatment.

Alongside a predictive performance that was better as compared to logistic regression, the decision-tree model provides richness to our study findings. The patient subgroups identified by recursive partitioning of the decision tree model enable clinicians and public health experts to better understand variation in treatment discontinuation rates among patients with OUD. Specifically, such recursive partitioning can be used to determine the thresholds that are most logical for dichotomizing features, which can then be used to reveal a valuable understanding of the potential interactions among the features. A set of decision classification rules can be formulated for patient profiles that are most highly associated with the risk of treatment discontinuation based on the most relevant interactions among features.

Thus, while treatment discontinuation was most common among patients who have relatively lower medication adherence in the early phase of treatment (i.e., early PDC), treatment discontinuation was also found to be associated with age, gender, urbanicity of assigned county, insurance type (i.e., supply side indicator), and a proxy for income status. These findings are also consistent with previous studies [20, 44–47], which reported that patients’ age, gender, and income status are associated with buprenorphine treatment discontinuation and other related outcomes such as medication assisted treatment (MAT) for substance use disorders (SUD), prevalence of opioid misuse, and overdose. Previous research has also reported that patients’ access to and quality of substance use disorder treatment differ between urban and rural areas [47, 48]. The potential interactions among these features as revealed by the decision tree model would not have been possible by the regression-based methods. Such interactions among features provide knowledge that clinicians, payers, and policy makers can use to design potential interventions and public health policies for reducing patients’ premature discontinuation from OUD treatment.

Despite these contributions, we recognize several limitations of our study that may also provide opportunities for future research. First, we solely used information of patients’ medication refill history from prescription claims to measure treatment discontinuation. As such it was not possible to track patients who discontinued refilling the buprenorphine prescriptions to switch to methadone or another type of treatment program for OUD. Second, while our sensitivity test for inclusion of a patient’s baseline clinical information from claims data did not show much predictive value for treatment discontinuation, the availability of more complete and reliable clinical data from patients’ electronic health record (EHR) may prove more valuable in the development of future models for predicting treatment discontinuation. Third, we acknowledge that income-stratification based on county may not be truly representative of patients’ actual socio-economic status as in some counties there exists considerable variation regarding family income. Moreover, because we did not have information about patients’ actual residence (i.e., county or zip code), we used prescribers’ county as a proxy. While, as noted, we found a very high match rate between patients’ and prescriber’s location based on county, this proxy will not always be accurate. Fourth, we also note that for patients who discontinued within the first three months following treatment initiation, there was overlap in the information used for determining PDC values and for discontinuation (i.e., two consecutive months without any refills). However, this applied only to approximately 20% of the total sample who discontinued within that time frame. Moreover, among those who did discontinue within that time frame, substantial variation existed for PDC values so that this feature still had predictive value for these patients. Specifically, the mean of the three-month PDC of those patients was 56% but the standard deviation was 35.6%. The remaining patients who did not discontinue within the first three months had a mean three-months PDC of 92.3% with a standard deviation of 20.7%.

In conclusion, machine learning models can help predict which patients will be at higher risk of discontinuing treatment for OUD. For this prediction, medication adherence at the early treatment phase added valuable information to patient-level demographic and prescriber-level characteristics that are generally available at treatment initiation. Thus, our proposed machine learning framework can serve as an initial step to developing an automated decision support tool that can be implemented in different clinical settings. Performing such prediction for treatment discontinuation can potentially help prescribers develop personalized support systems to improve patients’ long-term retention in OUD treatment. Prescribers can design personalized interventions that account for pharmacologic aspects of the treatment (e.g., choice of medication, dosage and duration of the medication), psychosocial adjuncts (e.g., counselling, cognitive behavioral therapy), contingency management, care coordination supports (e.g., peer recovery coach and navigators), financial support (e.g., medication/program reimbursement), social services and/or logistic supports (e.g., housing, transportation, day care), and information technology (e.g., self-management apps) [49]. While not all of these interventions will be suitable for every patient at higher risk of treatment discontinuation, the decision rules proposed in our study can help prescribers anticipate the support systems that will best meet the patients’ need for successful retention in the treatment.

## Data Availability

The datasets analyzed during the current study are not publicly available due to a data use agreement between authors and Center for Health Information and Analysis (CHIA). Information about the Massachusetts All Payer Claims Database (MA APCD) and the application process to request the data can be obtained from CHIA website (https://www.chiamass.gov/ma-apcd/) or via email (apcd.data@chiamass.gov).

## Appendix

See Tables 3 and 4.

**Table 3:** List of given grids of hyper-parameters and selected hyper-parameters used in the machine learning models for the prediction of buprenorphine treatment discontinuation

Treatment stage for making prediction	Machine learning model	Given hyperparameters	Selected hyperparameters
First stage models with baseline predictors	Logistic regression	solver: newton-cg, lbfgs, liblinear	solver: newton-cg
		penalty: l1, l2, elasticnet, none	penalty: none
		penalty/regularization strength, C: 0.001, 0.01, 0.1, 1, 10	C: 0.001
	Decision tree	criterion: gini, entropy	criterion: gini
		min_samples_leaf: 10, 20, 30, 40, 50	min_samples_leaf: 20
	Random forest	criterion: gini, entropy	criterion: gini
		min_samples_leaf: 5, 10, 20, 30, 40	min_samples_leaf: 20
		n_estimators: 100, 200, 300, 400, 500	n_estimators: 100
	Extreme gradient boosting	learning_rate: 0.0001, 0.001, 0.01, 0.1, 1	learning_rate: 1
		max_depth: 10, 20, 30, 40, 50	max_depth: 40
	Neural network	activation: relu, tanh, sigmoid, hard_sigmoid, linear	activation: relu
		neurons: 10, 50, 100	neurons: 100
		optimizer: SGD, RMSprop, Adagrad, Adadelta, Adam, Adamax, Nadam	optimizer: Nadam
		epochs: 1, 10	epochs: 10
		batch_size: 1000, 2000	batch_size: 1000
	Support vector machine	degree: 3, 4, 5, 6	degree: 3
		gamma: 0.001, 0.01, 0.1	gamma: 0.1
		C: 1, 10, 100	C: 10
Second stage models including 2 months PDC as continuous measure	Logistic regression	solver: newton-cg, lbfgs, liblinear	solver: liblinear
		penalty: l1, l2, elasticnet, none	penalty: l2
		penalty/regularization strength, C: 0.001, 0.01, 0.1, 1, 10	C: 0.1
	Decision tree	criterion: gini, entropy	criterion: entropy
		min_samples_leaf: 10, 20, 30, 40, 50	Min_samples_leaf: 40
	Random forest	criterion: gini, entropy	criterion: gini
		min_samples_leaf: 5, 10, 20, 30, 40	min_samples_leaf: 10
		n_estimators: 100, 200, 300, 400, 500	n_estimators: 200
	Extreme gradient boosting	learning_rate: 0.0001, 0.001, 0.01, 0.1, 1	learning_rate: 1
		max_depth: 10, 20, 30, 40	max_depth: 20
	Neural network	activation: relu, tanh, sigmoid, hard_sigmoid, linear	activation: linear
		neurons: 10, 50, 100	neurons: 100
		optimizer: SGD, RMSprop, Adagrad, Adadelta, Adam, Adamax, Nadam	optimizer: RMSprop
		epochs: 1, 10	epochs: 10
		batch_size: 1000, 2000	batch_size: 1000
	Support vector machine	degree: 3, 4, 5, 6	degree: 3
		gamma: 0.001, 0.01, 0.1	gamma: 0.1
		C: 1, 10, 100	C: 100
Second stage models including 3 months PDC as continuous measure	Logistic regression	solver: newton-cg, lbfgs, liblinear	solver: liblinear
		penalty: l1, l2, elasticnet, none	penalty: l2
		penalty/regularization strength, C: 0.001, 0.01, 0.1, 1, 10	C: 0.01
	Decision tree	criterion: gini, entropy	criterion: gini
		min_samples_leaf: 10, 20, 30, 40, 50	Min_samples_leaf: 40
	Random forest	criterion: gini, entropy	criterion: gini
		min_samples_leaf: 5, 10, 20, 30, 40	min_samples_leaf: 10
		n_estimators: 100, 200, 300, 400, 500	n_estimators: 100
	Extreme gradient boosting	learning_rate: 0.0001, 0.001, 0.01, 0.1, 1	learning_rate: 1
		max_depth: 10, 20, 30, 40	max_depth: 30
	Neural network	activation: relu, tanh, sigmoid, hard_sigmoid, linear	activation: tanh
		neurons: 10, 50, 100	neurons: 100
		optimizer: SGD, RMSprop, Adagrad, Adadelta, Adam, Adamax, Nadam	optimizer: Adam
		epochs: 1, 10	epochs: 10
		batch_size: 1000, 2000	batch_size: 1000
	Support vector machine	degree: 3, 4, 5, 6	degree: 3
		gamma: 0.001, 0.01, 0.1	gamma: 0.1
		C: 1, 10, 100	C: 100
Second stage models including 2 months PDC as categorical measure	Logistic regression	solver: newton-cg, lbfgs, liblinear	solver: liblinear
		penalty: l1, l2, elasticnet, none	penalty: l2
		penalty/regularization strength, C: 0.001, 0.01, 0.1, 1, 10	C: 0.01
	Decision tree	criterion: gini, entropy	criterion: gini
		min_samples_leaf: 10, 20, 30, 40, 50	Min_samples_leaf: 30
	Random forest	criterion: gini, entropy	criterion: gini
		min_samples_leaf: 5, 10, 20, 30, 40	min_samples_leaf: 10
		n_estimators: 100, 200, 300, 400, 500, 600	n_estimators: 500
	Extreme gradient boosting	learning_rate: 0.0001, 0.001, 0.01, 0.1, 1	learning_rate: 1
		max_depth: 10, 20, 30, 40	max_depth: 10
	Neural network	activation: relu, tanh, sigmoid, hard_sigmoid, linear	activation: tanh
		neurons: 10, 50, 100	neurons: 100
		optimizer: SGD, RMSprop, Adagrad, Adadelta, Adam, Adamax, Nadam	optimizer: RMSprop
		epochs: 1, 10	epochs: 10
		batch_size: 1000, 2000	batch_size: 2000
	Support vector machine	degree: 3, 4, 5, 6	degree: 3
		gamma: 0.001, 0.01, 0.1	gamma: 0.1
		C: 1, 10, 100	C: 10
Second stage models including 3 months PDC as categorical measure	Logistic regression	solver: newton-cg, lbfgs, liblinear	solver: liblinear
		penalty: l1, l2, elasticnet, none	penalty: l2
		penalty/regularization strength, C: 0.001, 0.01, 0.1, 1, 10	C: 0.1
	Decision tree	criterion: gini, entropy	criterion: gini
		min_samples_leaf: 10, 20, 30, 40, 50	Min_samples_leaf: 40
	Random forest	criterion: gini, entropy	criterion: entropy
		min_samples_leaf: 5, 10, 20, 30, 40	min_samples_leaf: 10
		n_estimators: 100, 200, 300, 400, 500, 600	n_estimators: 500
	Extreme gradient boosting	learning_rate: 0.0001, 0.001, 0.01, 0.1, 1	learning_rate: 1
		max_depth: 10, 20, 30, 40	max_depth: 40
	Neural network	activation: relu, tanh, sigmoid, hard_sigmoid, linear	activation: relu
		neurons: 10, 50, 100	neurons: 50
		optimizer: SGD, RMSprop, Adagrad, Adadelta, Adam, Adamax, Nadam	optimizer: RMSprop
		epochs: 1, 10	epochs: 10
		batch_size: 1000, 2000	batch_size: 2000
	Support vector machine	degree: 3, 4, 5, 6	degree: 3
		gamma: 0.001, 0.01, 0.1	gamma: 0.1
		C: 1, 10, 100	C: 10

**Table 4:** Buprenorphine treatment discontinuation prediction performance of machine learning models at first and second stage of treatment

Treatment stage for making prediction	Performance metrices	Decision tree	Random forest	Extreme gradient boosting	Logistic regression	Neural network	Support vector machine
First stage models with baseline predictors	Precision	0.57 ± 0.01	0.56 ± 0.02	0.53 ± 0.02	0.56 ± 0.02	0.56 ± 0.04	0.57 ± 0.03
	Recall	0.44 ± 0.02	0.49 ± 0.02	0.52 ± 0.03	0.38 ± 0.02	0.42 ± 0.05	0.38 ± 0.03
	F1 score	0.49 ± 0.02	0.53 ± 0.02	0.52 ± 0.03	0.45 ± 0.01	0.47 ± 0.03	0.38 ± 0.02
	C-statistics	0.58 ± 0.01	0.59 ± 0.02	0.55 ± 0.02	0.57 ± 0.02	0.57 ± 0.02	0.56 ± 0.02
Second stage models with 2 months PDC^*^ and baseline predictors	Precision	0.66 ± 0.02	0.67 ± 0.02	0.58 ± 0.02	0.76 ± 0.02	0.67 ± 0.07	0.66 ± 0.03
	Recall	0.50 ± 0.04	0.55 ± 0.01	0.57 ± 0.02	0.37 ± 0.01	0.51 ± 0.12	0.52 ± 0.01
	F1 score	0.57 ± 0.02	0.60 ± 0.01	0.58 ± 0.02	0.49 ± 0.01	0.55 ± 0.05	0.58 ± 0.01
	C-statistics	0.67 ± 0.01	0.69 ± 0.01	0.63 ± 0.02	0.67 ± 0.02	0.67 ± 0.02	0.64 ± 0.01
Second stage models with 3 months PDC^*^ and baseline predictors	Precision	0.67 ± 0.01	0.70 ± 0.02	0.62 ± 0.02	0.78 ± 0.03	0.72 ± 0.04	0.77 ± 0.06
	Recall	0.58 ± 0.04	0.59 ± 0.02	0.61 ± 0.01	0.44 ± 0.02	0.53 ± 0.03	0.44 ± 0.06
	F1 score	0.63 ± 0.02	0.63 ± 0.02	0.61 ± 0.01	0.56 ± 0.02	0.61 ± 0.02	0.56 ± 0.04
	C-statistics	0.71 ± 0.02	0.73 ± 0.02	0.68 ± 0.02	0.70 ± 0.02	0.70 ± 0.03	0.67 ± 0.02

*PDC is included in the model as a dichotomous variable

Each performance metric is reported as mean and standard deviation of the five values obtained from each of the five folds of cross validation

See Figs. 6, 7, 8.
